# Prospective Randomized Trial Comparing Endoscopic Sphincterotomy Followed by Surgery with Surgery Alone in Good Risk Patients with Choledocholithiasis

**DOI:** 10.1155/1996/64373

**Published:** 1996

**Authors:** R. Kapoor, S. P. Kaushik, V. A. Saraswat, G. Choudhuri, S. S. Sikora, R. Saxena, V. K. Kapoor

**Affiliations:** 1 Departments of Surgical Gastroenterology Sanjay Gandhi Post graduate Institute of Medical Sciences Lucknow 226014 India; 2 Departments of Medical Gastroenterology Sanjay Gandhi Post graduate Institute of Medical Sciences Lucknow 226014 India

## Abstract

*Background:* Role of endoscopic sphincterotomy (ES) in high risk patients with choledocholithiasis is established but its role in good risk patients is unclear.

* Design:* A prospective randomized trial of endoscopic sphincterotomy followed by surgery (ES + S) versus surgery alone (SA) in good risk patients with choledocholithiasis.

*Setting:* A tertiary level referral hospital in north India; July 1991 to October 1993.

*Patients and methods*: Thirty three out of 60 patients with choledocholithiasis were found suitable for randomization-16 were randomised to ES+S group and 17 to SA group.

*Results:* Common bile duct clearance was achieved in 11/13 (85%) patients in ES+S group and in 13/15 (87%) in SA group. Major complications occurred in 4/13 (31%) patients in ES+S group and 3/16 (19%) patients in SA group. These differences were not statistically significant, but patients in ES+S group were exposed to morbidity twice, procedure related morbidity of ES being 23%. No significant differences were observed in hospital stay and cost of treatment.

*Conclusions:*  Results of this trial do not support use of precholecystectomy ES in good risk patients with choledocholithiasis, since it did not offer any advantage over surgery alone.


*HPB Surgery*, 1996, Vol. 9, pp.145–148
Reprints available directly from the publisher
Photocopying permitted by license only

© 1996 OPA (Overseas Publishers Association)
Amsterdam B.V. Published in The Netherlands
by Harwood Academic Publishers GmbH
Printed in Malaysia

### Prospective Randomized Trial Comparing Endoscopic Sphincterotomy Followed by Surgery with Surgery Alone in Good Risk Patients with Choledocholithiasis

R. KAPOOR, S. P. KAUSHIK, V. A. SARASWAT,* G. CHOUDHURI,*
S. S. SIKORA, R. SAXENA and V. K. KAPOOR

Departments of Surgical Gastroenterology and Medical Gastroenterology *
Sanjay Gandhi Post graduate Institute of Medical Sciences, Lucknow 226014, India

*Background:* Role of endoscopic sphincterotomy (ES) in high risk patients with choledocholithiasis is established but its role in good risk patients is unclear.
*Design:* A prospective randomized trial of endoscopic sphincterotomy followed by surgery (ES + S) versus surgery alone (SA) in good risk patients with choledocholithiasis.
*Setting:* A tertiary level referral hospital in north India; July 1991 to October 1993.
*Patients and methods:* Thirty three out of 60 patients with choledocholithiasis were found suitable for randomization – 16 were randomised to ES+S group and 17 to SA group.
*Results:* Common bile duct clearance was achieved in 11/13 (85%) patients in ES+S group and in 13/15 (87%) in SA group. Major complications occurred in 4/13 (31%) patients in ES+S group and 3/16 (19%) patients in SA group. These differences were not statistically significant, but patients in ES+S group were exposed to morbidity twice, procedure related morbidity of ES being 23%. No significant differences were observed in hospital stay and cost of treatment.
*Conclusions:* Results of this trial do not support use of precholecystectomy ES in good risk patients with choledocholithiasis, since it did not offer any advantage over surgery alone.

**KEY WORDS:** Cholelithiasis common bile duct calculi sphincterotomy endoscopic retrograde cholangiopancreatography.

### INTRODUCTION

Endoscopic sphincterotomy (ES) has gained wide and universal acceptance for management of choledocholithiasis in postcholecystectomy patients and in high risk patients with gallbladder (GB) in situ. ES has also become the treatment of choice in patients with acute cholangitis and pancreatitis due to gallstones.[1] However, no clear guidelines are available regarding the role of ES in good risk patients with choledocholithiasis and GB in situ. Endoscopic removal of the common bile duct (CBD) stones followed by cholecystectomy as an alternative to GB and CBD surgery has not been properly evaluated so far. Conflicting reports have appeared supporting[2,3] as well as opposing[4,5] routine use of ES in these patients.

We conducted a prospective randomized trial comparing ES followed by surgery with surgery alone in good risk patients with choledocholithiasis and report the results.

*Correspondence to*: Prof S.P. Kaushik, Head of the Department of Surgical Gastroenterology, Sanjay Gandhi Post-graduate Institute of Medical Sciences, Lucknow 226 014, India. Fax 91 (522) 259973

### PATIENTS AND METHODS

Between July 1991 and October 1993, 420 patients with gallstone disease were seen in the Department of

Surgical Gastroenterology at the Sanjay Gandhi Post-Graduate Institute of Medical Scienes, Lucknow – a tertiary level referral hospital in north India where gallstone disease in common. Sixty out of these 420 patients were suspected to have choledocholithiasis after clinical history and examination, biochemical tests and ultrasonography (US). The suspicion of choledocholithiasis was based on the presence of one or more of the following criteria: total serum bilirubin greater than 34.2 umol/L ($n = 8$), serum alkaline phosphatase greater than 235 IU/L (normal 35–125 IU/L) ($n = 18$), CBD diameter greater than 10 mm ($n = 18$) and probable CBD stone ($n = 25$) on US.

All 60 patients underwent endoscopic retrograde cholangiopancreaticography (ERCP), under cover of prophylactic antibiotics, before randomization. The predetermined exclusion criteria were: i) elderly and frail patient ($n = 1$), ii) unsuitability to undergo major surgery ($n = 0$), iii) pregnancy ($n = 0$), iv) patients presenting with acute cholangitis and septicaemia ($n = 9$), v) failure to cannulate the CBD at ERCP ($n = 3$), and vi) stone larger than 15 mm ($n = 12$). Two patients were found not to have CBD stones on ERCP and were excluded from the trial. A total of 27 patients were thus excluded.

Following ERCP, the remaining 33 patients were randomized into two groups by drawing sealed envelopes that contained computer-generated random assignments. In the ES+S group, ES was undertaken at the same time as ERCP. Clearance of the CBD was achieved by basket retrievel or by subsequent spontaneous passage of calculi and a clear CBD was documented in all patients prior to surgery. Surgery in these patients was scheduled on elective basis within 6 weeks after ES. In the SA group, surgery was undertaken on the next available operating list. CBD exploration was performed in conventional fashion; choledochoscopy ($n = 6$) was employed at the discretion of the operating surgeon.

The two groups were compared according to clinical features and biochemical parameters (Table 1). Cases were analyzed according to 'intention to treat' policy. Outcome in every case was assessed in terms of mortality, morbidity (major and minor), length of hospital stay and the overall cost of treatment. Information about hospital charges was obtained directly from the hospital finance office while details of additional expenditure on drugs etc were taken from the patients or their attendants.

Fisher's exact test was used for comparison of clinical parameters, success of treatment and morbidity. Student's t test was used to compare haematological and biochemical parameters and hospital stay and cost of treatment.

**Table 1** Comparison of clinical features and biochemical parameters in two groups

| | *ES + S group (n = 13)* | | *SA group (n = 16)* | | *P value* |
|---|---|---|---|---|---|
| *Clinical features* | | | | | |
| Age (Years)* | 42 | (20–60) | 46 | (24–75) | 0.55 |
| Pain (%) | 13 | (100%) | 15 | (94%) | 1.00 |
| Jaundice (%) | 5 | (38%) | 4 | (25%) | 0.68 |
| Fever (%) | 4 | (31%) | 4 | (25%) | 1.00 |
| Acute cholangitis (%) | 4 | (31%) | 4 | (25%) | 1.00 |
| *Biochemical parameters** | | | | | |
| Hb (g/L) | 114 | (90–130) | 108 | (72–134) | 0.11 |
| TLC × $10^3$/cumm | 7.7 | (3.3–12.0) | 7.4 | (3.5–12.0) | 0.36 |
| Bilirubin (umol/L) | 31 | (6.8–118) | 12 | (3.4–111) | 0.22 |
| SAP (IU/L) | 255 | (57–978) | 300 | (57–650) | 0.46 |
| SGOT (IU/L) | 50 | (11–292) | 57 | (08–212) | 0.29 |
| SGPT (IU/L) | 64 | (11–276) | 58 | (19–213) | 0.49 |
| Albumin (g/L) | 40 | (27–50) | 42 | (35–52) | 0.18 |
| Creatinine (umol/L) | 80 | (53–106) | 80 | (53–124) | 0.24 |

* mean (range).
ES+S - endoscopic sphincterotomy followed by surgery, SA-surgery alone, Hb- haemoglobin, TLC -total leucocyte count, SAP - serum alkaline phosphatase, SGOT-serum glutamate oxalate transaminase, SGPT-serum glutamate pyruvate transaminase.

### RESULTS

Thirty three out of 60 (55%) patients with choledocholithiasis could be entered into the trial. Sixteen patients were randomized to ES+S group and 17 to SA group. The two groups were comparable with respect to clinical features and biochemical parameters (Table I).

### *ES+S Group (n=16)*

Thirteen patients completed treatment; three patients were excluded from analysis because two did not complete treatment and one was found to have carcinoma of the gall bladder at surgery.

Successful clearance of the CBD was achieved in 11/13 (85%) patients. Elective cholecystectomy alone was performed in 10 patients. Cholecystectomy with CBD exploration was performed in three patients – in two patients because of failure of ES to clear the CBD and in one patient who refused to undergo check ERCP since he had bleeding following ES.

The overall morbidity was 5/13 (39%) – major morbidity occurred in three patients (23%) following ES. Two patients had acute cholangitis, one of whom also had septicaemia and major upper gastrointestinal bleed and one had an attack of acute pancreatitis. All complications were managed successfully by conservative measures. Major morbidity after elective cholecystectomy occurred in two patients who had chest infection and major wound infection; one of these patients had acute cholangitis after ES while the other had uneventful ES. Major complications thus occurred in 4/13 (31%) patients. Minor complications occurred in 1/13 (8%) patient. The mean length of hospital stay was 10.6 days (range 6–18 days) but five patients needed admission twice for completion of treatment. The number of days between ES and cholecystectomy varied from 1 to 47 days with median of 20 days. The mean cost of overall treatment was Rupees 4,748. (US $ 148 approximately).

*SA Group (n=17)*

Sixteen patients completed treatment. One patient who was found to have carcinoma of the gall bladder at surgery was excluded. CBD was explored in 15 patients; one patient developed actue cholecystitis and empyema of the gall bladder following ERCP and underwent emergency cholecystostomy. On subsequent cholecystocholangiogram 8 weeks later CBD was found to be stone free and hence he did not require CBD exploration.

Complete duct clearance was achieved in 13/15 (87%) patients. Two (13%) patients had retained stones which were later managed successfully with ES.

The overall morbidity was 5/16 (31%). Three patients (19%) developed major complications (acute cholangitis, chest infection, intraoperative ventricular tachycardia and hypotension). Minor complications occurred in 2/16 (12%) patients. The mean length of hospital stay was 11.3 days (range 6–24 days). Three patients were operated during their second admission. The mean cost of over all treatment was Rupees 4,305. (US $ 135 approximately).

Table 2 gives a comparative summary of the results in the two groups.

**Table 2** Results in two groups

| | *ES+S group* | *SA group* |
|---|---|---|
| No of patients | 16 | 17 |
| No of patients who completed treatment | 13 | 16 |
| No of patients analysed | 13 | 15 |
| CBD cleared | 11 (85%) | 13 (87%) |
| Morbidity | 5 (39%) | 5 (31%) |
| major | 4 (31%) | 3 (19%) |
| Hospital stay (days) | 10.6 (6–18) | 11.3 (6–24) |
| No of patients requiring second admission | 5 (39%) | 3 (19%) |
| Cost of treatment | Rs 4,748 (US $ 148) | Rs 4,305 (US $ 135) |

ES + S - endoscopic sphicterotomy followed by surgery; SA - surgery alone; Rs - Rupees.

### DISCUSSION

This prospective randomised trial of endoscopic sphincterotomy followed by surgery (ES+S) versus surgery alone (SA) in good risk patients with choledocholithiasis did not reveal any significant difference in duct clearance, morbidity, hospital stay and cost between the two groups.

Successful management of CBD stones by ES in a select group of patients e.g. postcholecystectomy and high risk patients has prompted some workers[2,3] to try its use for CBD clearance on a routine basis. Retrospective analysis of data from the Virginia Mason Medical Center[6] and the University Hospital of Innsbruck,[7] however, indicated no significant difference in morbidity or mortality in patients managed endoscopically or surgically. An increased incidence of retained CBD stones was seen after ES as compared to the surgically treated group; this difference achieved statistical significance in the report form the University Hospital of Innsbruck[7].

Neoptolemos *et al.*[4] in a prospective randomized trial of ES for clearance of CBD stones prior to open cholecystectomy versus one time surgical treatment, found an overall complication rate of 33% and major complication rate of 16% in the preoperative ES group, as compared to 22% and 9% respectively in the surgery alone group. Though the differences did not achieve significance, a trend towards higher complication rates in the ES group was noted. The study was stopped since it was felt that routine preoperative ES had not offered any significant advantage over conventional therapy.

In another study by the same authors,[8] a higher complication rate was again found in patients undergoing preoperative ES. Based on multivariate analysis of risk factors, these investigators recommended that good risk patients should continue to be treated by one time surgery and preoperative ES should be reserved for high risk patients only.

The present trial has also shown that the specific complications of preoperative ES were of a serious nature. The overall morbidity in these patients was greater than those who underwent one time surgical treatment, though the diVerences did not reach significance levels. Patients in the ES+S group were however exposed to procedure related morbidity twice and waited a mean of 20 days before having final treatment i.e. cholecystectomy. Study design and predetermined exclusion criteria resulted in as many as 27 (45%) patients being excluded from the trial 12 of these because the stone size was greater than 15 mm. One time surgical option on the other hand would have been feasible in all of them. The factors which excluded use of preoperative ES thus in no way precluded the institution and/or the outcome of one time surgical treatment.

Support to our point of view has also come from Stain *et al.*[5] who, based on efficacy, morbidity and cost, did not find preoperative ES for removal of the CBD stones to be better than one time surgery. Similar observations were made by Stiegmann *et al.*[9] who showed that there was no added advantage of precholecystectomy ES in such patients.

The present study did not address the question of preoperative ES in patients being taken up for laparoscopic cholecystectomy. There is no consensus in the available literature on this issue.[10–14] Some of the recent publications[15–17] however do not envisage a great role for preoperative ES in this setting.

In conclusion, this trial did not support the routine use of precholecystectomy ES in good risk patients with choledocholithiasis. This was because in the precholecystectomy ES group, selection of patients became mandatory on the basis of past experiences and defined norms to ensure successful outcome of ES; because patients in the ES+S group were exposed to procedure related morbidity twice; and because the overall results in the ES+S group did not prove to be better than those in the surgery alone group. More over, the universal applicability of surgical intervention as compared to selective application of ES remains unmatched.

### REFERENCES

1. Summerfield A. Biliary obstruction is best managed by endoscopists. *Gut*, 1988; **29**: 741–5.
2. Heinerman PM, Boeckl O, Pimpl W. Selective ERCP and preoperative stone removal in bile duct surgery. *Ann Surg*, 1989; **209**: 267–71.
3. Stiegmann GV, Pearlman NW, Goff JS, *et al.* Endoscopic cholangiography and stone removal prior to cholecystectomy. A more cost-effective approach than operative duct exploration? *Arch Surg* 1989; **124**: 787–90.
4. Neoptolemos JP, Carr-Locke DL, Fossard DP. Prospective randomized study of preoperative endoscopic sphincterotomy versus surgery alone for common bile duct stones. *Br Med J* 1987; **294**: 470–4.
5. Stain SC, Cohen H, Tsuishoysha M, Donovan AJ. Choledocholithiasis: endoscopic sphincterotomy or common bile duct exploration. *Ann Surg* 1991; **213**: 627–34.
6. Miller BM, Kozarck RA, Ryan JA, Ball TJ, Traverso LW. Surgical versus endoscopic management of common bile duct stones. *Ann Surg* 1988; **207**: 135–41.
7. Schwab G, Pointner R, Wetscher G, Glaserk, Fortin E, Bodner E. Treatment of calculi of the common bile duct. *Surg Gynecol Obstet* 1992; **175**: 115–20.
8. Neoptolemos JP, Shaw DE, Carr-Locke DL. A multivariate analysis of preoperative risk factors in patients with common bile duct stones: implications for treatment. *Ann Surg* 1989; **209**: 157–61.
9. Steigmann GV, Gongh JS, Mansour A, Pearlman NW, Reveille RM, Nortan L. Precholecystectomy endoscopic cholangiography and stone removal is not superior to cholecystectomy, cholangiography and common duct exploration. *Am J Surg* 1992; **163**: 227–30.
10. Fink AS. To ERCP or not to ERCP? That is the question. *Surg Endosc* 1993; **7**: 375–6.
11. Martin IG, Curley P, McMahon MJ. Minimally invasive treatment for common bile duct stones. *Br J Surg* 1993; **80**: 103-6.
12. Graham SM, Flowers JL, Scott TR, et al. Laparoscopic cholecystectomy and common bile duct stones. The utility of planned preoperative endoscopic retrograde cholangiography and sphincterotomy: Experience with 63 patients. *Ann Surg* 1993; **218**: 61-7.
13. Brodish RJ, Fink AS. ERCP, cholangiography and laparoscopic cholecystectomy. The Society of American Gastrointestinal Endoscopic Surgeons (SAGES) opinion survey. *Surg Endosc* 1993; **7**: 3–8.
14. Fink AS. Current dilemmas in management of common duct stones. *Surg Endosc* 1993; **7**: 285–91.
15. Berci G. Preoperative ERCP and intraoperative cholangiography in the age of laparoscopic cholecystectomy. *Surg Endosc* 1993; **7**: 2.
16. Phillips EH. ERCP in conjuction with LC – Invited commentary. *Surg Endosc* 1993; **7**: 393–4.
17. Pitt HA. Role of open choledochotomy in the treatment of choledocholithiasis. *Am J Surg* 1993; **165**: 483–6.

